# Attentional processes in response to emotional facial expressions in adults with retrospectively reported peer victimization of varying severity: Results from an ERP dot-probe study

**DOI:** 10.1186/s40359-024-01958-5

**Published:** 2024-08-29

**Authors:** Klara Blauth, Benjamin Iffland

**Affiliations:** Department of Psychology, Bielefeld University, Postbox 100131, 33501 Bielefeld, Germany

**Keywords:** Peer victimization, Attentional bias, Dot-probe task, Event-related potentials, Emotional facial expressions

## Abstract

**Background:**

Attentional processes are influenced by both stimulus characteristics and individual factors such as mood or personal experience. Research has suggested that attentional biases to socially relevant stimuli may occur in individuals with a history of peer victimization in childhood and adolescence. Based on this, the present study aimed to examine attentional processes in response to emotional faces at both the behavioral and neurophysiological levels in participants with experiences of peer victimization.

**Methods:**

In a sample of 60 adult participants with varying severity of retrospectively reported peer victimization in childhood and adolescence, the dot-probe task was administered with angry, disgusted, sad, and happy facial expressions. In addition to behavioral responses, physiological responses (i.e., event-related potentials) were analyzed.

**Results:**

Analyses of mean P100 and P200 amplitudes revealed altered P200 amplitudes in individuals with higher degrees of peer victimization. Higher levels of relational peer victimization were associated with increased P200 amplitudes in response to facial expressions, particularly angry and disgusted facial expressions. Hierarchical regression analyses showed no evidence for an influence of peer victimization experiences on reaction times or P100 amplitudes in response to the different emotions.

**Conclusion:**

Cortical findings suggest that individuals with higher levels of peer victimization mobilize more attentional resources when confronted with negative emotional social stimuli. Peer victimization experiences in childhood and adolescence appear to influence cortical processes into adulthood.

**Supplementary Information:**

The online version contains supplementary material available at 10.1186/s40359-024-01958-5.

## Background

People perceive stimuli in their environment with varying intensity and speed. Shifting attention toward or away from certain stimuli allows for filtering of environmental stimuli and is influenced by the individual relevance of a stimulus (e.g., [1]). Following this, studies of attentional processes in healthy populations show that emotional, and especially potentially threatening stimuli, elicit altered attentional processes compared to neutral stimuli [2, 3]. Accordingly, attentional biases towards threatening stimuli may, on the one hand, reflect increased vigilance for relevant stimuli. In this context, an increased vigilance represents an adaptive modification, as it allows people to respond to the most relevant stimuli [1, 4]. On the other hand, some studies have found attentional avoidance in non-clinical populations, which has been interpreted as a strategy for regulating emotions in response to mildly threatening, non-action-relevant stimuli, and is therefore thought to be useful in the context of mood regulation [2, 3]. Thus, attentional processes are shaped by stimulus characteristics, such as emotional content, and individual aspects, and can in turn influence both the perception and interpretation of stimuli or a situation. Attentional processes can be studied experimentally by analyzing both reaction times and cortical responses. Since attended stimuli elicit a more pronounced amplitude than unattended stimuli, the analysis of event-related potentials is suitable for the analysis of attentional processes [5]. In this context, early visual components such as the occipital P100 and P200 were considered relevant as they reflect basal and attentional processing with sensitivity to faces and emotional content [6–10].

Attentional biases, which are found in certain groups of people, reflect the relevance of a particular stimulus to an individual and the resulting changes in attentional processes. Studies in anxious populations show evidence of attentional biases associated with potentially threatening stimuli (for reviews, see [11, 12]). Evidence of altered attentional processes associated with negative stimuli has also been found in depressed samples (for an overview, see [13]). Because these biases toward threatening or mood-congruent stimuli can influence perception and behavior, attentional biases are thought to be relevant to the development and maintenance of mental illness (for review, see [12]). In addition to an association between attentional biases and psychopathology, study findings also suggest altered attentional processes in individuals with experiences of maltreatment in childhood and adolescence, both by caregivers (i.e., child maltreatment; e.g., [14–18]) and in the peer context (i.e., peer victimization; e.g., [19, 20]).

Experiences of relational peer victimization in childhood and adolescence refer to experiences in which individuals are excluded from a peer group, experience rejection, or are ignored by peers [21, 22]. Studies have shown that experiences of peer victimization are associated with lower social functioning and greater risk for several mental disorders, including anxiety, depression, or substance use disorders [23–25]. More frequent experiences of peer victimization have been shown to be associated with more severe outcomes in terms of psychopathology [24, 25]. These findings have led numerous studies to address possible processes that may mediate between peer victimization experiences and psychopathology, and thus attentional processes have been examined at both the behavioral and cortical levels in individuals with experiences of peer maltreatment [19, 20, 26, 27]. At the behavioral level, altered attentional processes related to negative or potentially threatening stimuli have been found in the context of peer victimization [19, 20]. After applying a social conditioning task, Iffland and Neuner [20] analyzed reaction times in a dot-probe task as well as an emotional Stroop task and found altered attentional processes in response to neutral faces that were previously conditioned negatively and neutrally in participants with higher levels of peer victimization experiences. However, behavioral findings are inconclusive, as both attentional avoidance of threatening stimuli (e.g., [19]) and faster orienting processes to negative stimuli (e.g., sad stimuli; [28]) have been suggested in peer victimized participants. Stimulus choice and paradigm appear to have a critical influence on behavioral outcomes in the context of peer victimization experiences.

Subsequently, analysis of event-related potentials (ERPs) provides a more detailed analysis of attentional processes. In the context of maltreatment, studies indicate an altered P100 amplitude in affected individuals [29–31]. Using a selective attention paradigm, Pollak and Tolley-Schell [31] found evidence of increased P100 amplitude in response to angry faces in children with a history of physical violence. Combined with faster reaction times on valid anger trials, the results suggest an increased salience of anger in children with experiences of physical abuse, which may subsequently lead to a facilitated motor response [31]. Following the findings of P100 amplitude changes in maltreated individuals, Iffland et al. [26] found an increased P100 amplitude in response to previously negative and neutral conditioned neutral faces in depressed participants with a history of relational peer victimization, which was interpreted as a sign of increased selective attention to interpersonal information [26]. Although this contrasts with the finding of no group differences in P100 amplitude in response to emotional faces in young children with experiences of violence [32], the various study findings suggest a more pronounced P100 amplitude in people with a history of maltreatment. In addition to the P100, the P200 has also been associated with early visual attention and sensitivity to the emotional content of a stimulus [33–36]. Studies suggest P200 modulation in the presentation of basic stimuli and in the anticipation and feedback processing of social stimuli, indicating an increased amplitude with respect to relevant stimuli [37–39]. Rossignol et al. [39] found an increased occipital P200 component in response to anger-neutral cues compared to fear-neutral cues in individuals with high levels of social anxiety. Here, the more pronounced amplitude was interpreted to be associated with an increased use of attentional resources in response to negative information with high motivational salience [39]. The results suggest that the P200 amplitude is associated with hypervigilance and increased allocation of attentional resources to potentially threatening stimuli. In addition, recent findings suggest a modulation of the P200 amplitude in peer victimized participants. Panier et al. [40] found that adolescents who reported higher levels of peer victimization showed a reduced P200 amplitude in response to social reward. The P200 may be a promising ERP component in the context of peer victimization and social stimuli, given the sensitivity of the P200 amplitude in the context of individual relevance and recent evidence for altered amplitudes in peer victimized participants.

As previous reports on behavioral measures in particular are ambiguous and partly contradictory, the current study aims to extend the existing literature on attentional biases by including the assessment of cortical processes (i.e., event-related potentials). Physiological studies suggest an increased sensitivity of cortical processes to social stimuli in certain groups of participants. Following initial findings on the relationship between peer victimization and cortical processes implicating differences in the perception and processing of stimuli, the present study focuses on the perception and processing of social stimuli. Thus, the present study is directly related to initial findings on altered processing of emotional [19] or social stimuli [20, 26] in peer victimized participants and should allow for a more differentiated analysis of attentional processes in relation to different emotional social stimuli. The aim is to find out whether attentional processes related to relevant social information, such as certain emotional facial expressions, change with increasing experience of peer victimization.

To examine the relationship between peer victimization experiences and altered attentional processes at the behavioral and cortical levels, the facial dot-probe task was used [3]. For this, emotional facial expressions were combined with neutral ones. Emotional faces, including happy, angry, disgusted, and sad faces, were used to investigate attentional processes to positive as well as socially negative (i.e., angry, and disgusted faces) and non-socially negative (i.e., sad faces) stimuli. Angry and disgusted facial expressions were considered relevant to peer victimization experiences because these emotions can be interpreted as meaningful about one’s relationship with others and possible (potentially threatening) actions by others. Thus, angry facial expressions have an interpersonal character, as they can provide information about the behavioral tendencies of the counterpart [41], as well as disgusted facial expressions, as they can be triggered to ensure social order [42, 43]. At the behavioral level, we expected an attentional bias toward potentially threatening stimuli (angry and disgusted faces) in participants with higher levels of experienced peer victimization. At the cortical level, based on findings related to maltreatment and social exclusion [31, 39], we expected a more pronounced mean amplitude for the P100 and P200 in individuals with more reported peer victimization experiences. As with the behavioral measures, we expected to find this hypervigilance in response to potentially threatening stimuli (angry and disgusted faces) in the context of peer victimization experiences, whereas the behavioral and cortical response to positive and sad facial expressions should be unaffected by the level of peer victimization experienced.

## Methods

### Participants

Participants were recruited through social media advertisements, flyers, and bulletins. In the laboratory, participants were informed about the conditions of participation, the procedure of an electroencephalography (EEG) measurement, and the financial compensation for participation. Each participant was required to give prior informed consent to participate in the study. Inclusion criteria were an age between 18 and 65 years and sufficient knowledge of the German language to understand the questionnaires and instructions. In addition, normal vision was required to participate in the study. There were no other exclusion criteria. The procedure was approved by the Ethics Committee of Bielefeld University.

Information on general sociodemographic variables such as age, gender, education level, and marital status was requested, as well as potentially relevant variables in the context of the analysis of physiological data, such as smoking, alcohol consumption, presence of epilepsy, and medication. A total of 67 people participated in the study and data from 60 people were included in the final sample. The relevant sociodemographic and psychopathological characteristics of the 60 participants included in both the behavioral and physiological analyses are presented in Table 1.

**Table 1:** Subject sociodemographic and psychopathological characteristics of the final sample (*N*=60)

Characteristics	
Gender, % female (*n*)	56.7 (34)
Age, *M* (*SD*), range	31.22 (11.83), 19-64
Family status, % single (*n*)	35 (21)
Educational status (high school or higher), % (*n*)	86.6 (52)
Mental disorder ever in lifetime^a^, % (*n*)	15 (9)
Symptoms of depression^b^, *M* (*SD*), range	2.95 (3.19), 0-13
Psychopathology^c^, *M* (*SD*), range	8.22 (7.99), 0-35
Trait Anxiety^d^, *M* (*SD*), range	33.2 (8.28), 21-56
Child maltreatment experiences^e^, *M* (*SD*), range	33.27 (7.91), 25-65
Peer victimization experiences^f^, *M* (*SD*), range	6.17 (4.10), 1-17

*Note.* ^a^based on self-report; ^b^Beck Depression Inventory; ^c^Symptom Checklist-27; ^d^State Trait Anxiety Inventory (Trait); ^e^Childhood Trauma Questionnaire; ^f^Fragebogen zu belastenden Sozialerfahrungen (Adverse Social Experiences Questionnaire)

### Instruments

#### Symptoms of psychopathology

General symptoms of psychopathology were assessed using the Symptom Check List-27 (SCL-27; [44]). Six subscales measure depressive, dysthymic, vegetative, agoraphobic, sociophobe symptoms, and symptoms of mistrust with a total score ranging from 0 to 108. In the present sample, the Symptom Check List-27 showed good internal consistency (Cronbach’s $\alpha$ = .90).

To assess current depressive symptomatology over the past two weeks the German version of the Beck Depression Inventory (BDI II; [45, 46]) was used. The BDI II assesses the presence and severity of depressive symptoms using 21 items on a scale from zero (absent) to three (severely present). Based on the total score with a range from 0 to 63, symptom severity can be classified (no/minimal, mild, moderate, or severe depressive symptoms). For this sample, an acceptable internal consistency was found (Cronbach’s $\alpha$ = .76).

Trait anxiety was assessed using the trait subscale of the State-Trait-Anxiety-questionnaire (STAI; [47, 48]). There are 20 items with a scale from one (almost never) to four (almost always), resulting in a total score with a range from 20 to 80. Here, the STAI had excellent internal consistency (Cronbach’s $\alpha$ =.91).

#### Experiences of child maltreatment and peer victimization

To examine experiences of relational peer victimization the Fragebogen zu belastenden Sozialerfahrungen (FBS (Adverse Social Experiences Questionnaire); [21]) was used. Different forms of relational peer victimization experiences that occurred during childhood (ages 6-12) and adolescence (ages 13-18) were measured retrospectively. There were 20 items in which participants were asked to indicate whether they had the experience in the first, second or both age periods. There is evidence that combining the two subscale scores into a total score is superior to subscales in capturing peer victimization experiences [21]. Therefore, this total score was used in all analyses. The total score can therefore range from 0 to 40. The questionnaire mainly asks about relational peer victimization, e.g. ’No one wanted to sit next to me in class’ or ’Other children or young people excluded me from their games or activities’. In this sample, the FBS showed an acceptable internal consistency (Cronbach’s $\alpha$ = .76).

To assess retrospective experiences of child maltreatment, the German version of the Childhood Trauma Questionnaire (CTQ; [49]) was used. On five subscales with 28 items in total, the questionnaire measures experiences of maltreatment (physical abuse, physical neglect, emotional abuse, emotional neglect, and sexual abuse). Since the items are to be rated on a scale from 1 (never true) to 5 (very often true), the possible values for the total score can range from 25 to 125. The total score for the CTQ showed good internal consistency (Cronbach’s $\alpha$ =.86).

### Paradigm and procedure

**Fig. 1:**
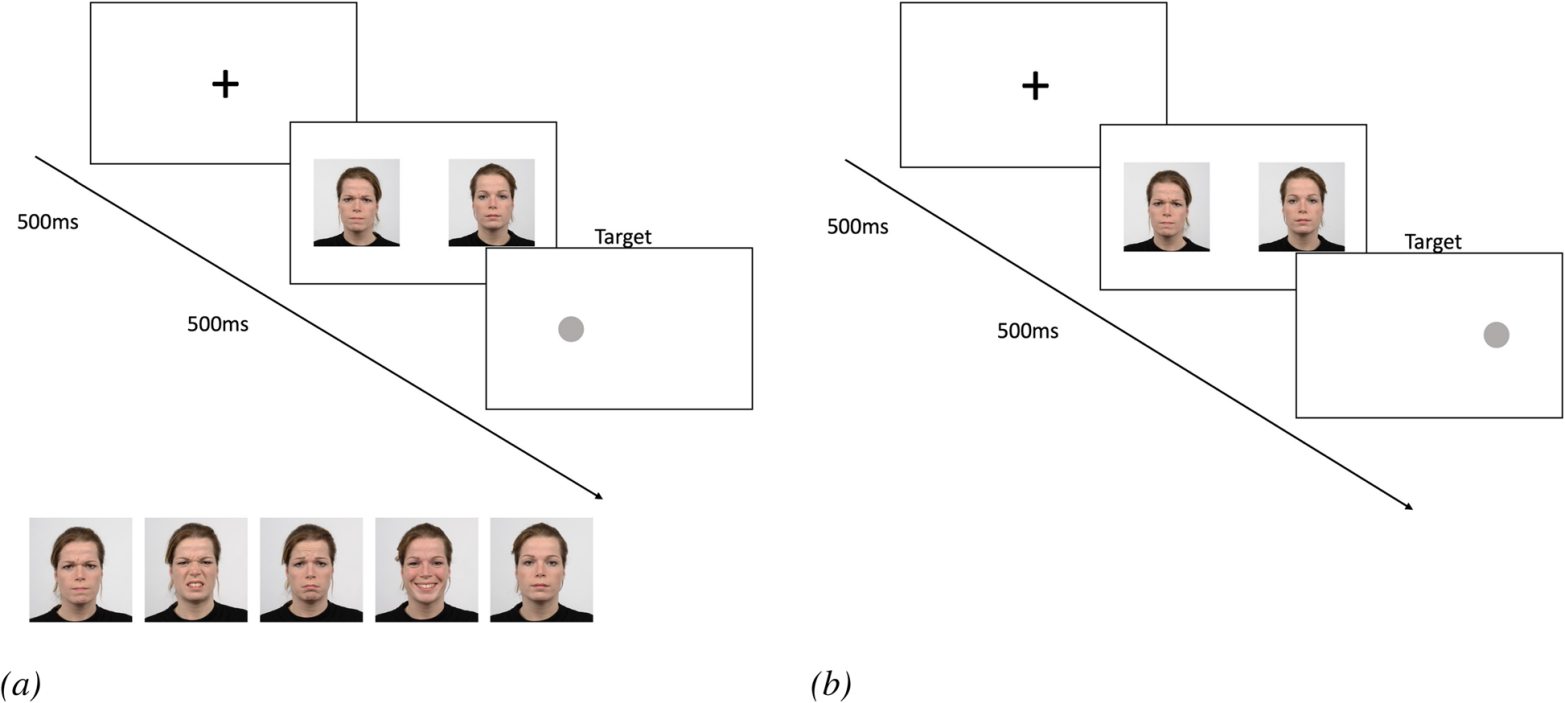
The experimental procedure for **a** congruent trials, including an overview of the valences included, and **b** incongruent trials

The facial dot-probe task was used to examine attentional processes [3]. Inquisit 6 (Millisecond software) was used to carry out the experimental procedure. Each trial began with a fixation cross presented in the center of the screen for 500 ms. This fixation cross was then replaced by two images presented simultaneously for 500 ms (one on the right, one on the left side of the screen). There were both emotion-neutral trials (i.e., EN trials) and neutral-neutral trials (i.e., NN trials). In EN trials (80% of all trials), one of the two pictures was emotional (i.e., emotional facial expression) and the other was neutral (i.e., neutral facial expression). Thus, each emotion (anger, disgust, sadness, happiness) was presented in 20% of all trials. In NN trials (20% of all trials), both presented pictures were neutral. The presentation of the pictures was followed by the presentation of a gray dot. In EN trials, this dot replaced either the emotional stimulus (congruent trials) or the neutral stimulus (incongruent trials). In NN trials, the dot always replaced one of the two neutral pictures. In both types of trials, trial presentation was randomized and equally distributed across all combinations. The participants’ task was to indicate on which side (right or left) the dot was to be seen by pressing the ’E’ key for left and the ’I’ key for right. The dot was presented on the left side of the screen in 50% of all trials and on the right side of the screen in the other 50% of all trials. The emotional facial expressions consisted of four different emotions, each paired with neutral faces (sad, happy, angry, and disgusted facial expressions). The order of the trials as well as the selection of the individual emotions was randomized. The stimulus material was taken from the Radboud Faces Database [50]. A total of 50 pictures of 10 actors (five women, five men) were used, each actor with each emotional expression was presented four times (2 blocks of 100 trials). For each trial, two images of the same actor were used. Trials with angry and disgusted faces were combined for all further analyses (anger/disgust trials), so that there were three different EN trials in addition to the NN trials (anger/disgust trials, sadness trials, and happiness trials) (Fig. 1).

### Data reduction and statistical analysis of behavioral data

As a BDI II score of 14 or more is considered to indicate mild depression [45], all participants with a BDI II total score greater than 13 were excluded from the analyses (*n*=1). The reaction time data of the participants included in the analyses were adjusted in several successive steps, following the procedure of other studies [20, 51, 52]. All trials in which the probe position was misinterpreted (1.21% of all trials) were excluded from the reaction time data. Furthermore, all trials with a reaction time of less than 150ms or more than 2000ms were excluded (0.02% of all valid trials). No participant’s mean deviated more than 3 *SD* from the mean reaction time. In addition, all trials in which the reaction time deviated more or less than 2 *SD* from the average reaction time of each subject were excluded from the analyses (2.28% of all trials). For the analysis of attentional bias, three different index scores were calculated for each emotion (attentional bias score, orienting score, and disengaging score; [52]). To calculate the attentional bias score, reaction times in congruent trials were subtracted from reaction times in incongruent trials. In this process, attentional biases toward the emotional stimulus are revealed by positive scores; attentional biases toward the neutral stimuli would be revealed by negative scores. In order to allow for more differentiated analyses, the orienting score was calculated in addition to the attentional bias score. For this purpose, reaction times for congruent EN trials are subtracted from NN trials. This allows conclusions to be drawn about whether individuals shift their attention to the emotional stimulus more quickly. In addition, to investigate whether individuals have difficulties disengaging their attention from the emotional stimulus, reaction times on NN trials were subtracted from incongruent EN trials to calculate the disengagement score.

### EEG recording and analyses

To record the EEG, 128 active electrodes were used (BioSemi Active Two System; www.biosemi.com) with a Sampling Rate of 512 Hz. The positioning of the 128 electrodes was done according to the BioSemi position system, which was ensured by positions given by a cap. Furthermore, two reference electrodes (CMS; common mode sense active electrode, DRL; driven right leg passive electrode) were used (www.biosemi.com/faq/cms&drl.htm). EMG (VEOG and HEOG) electrodes and ECG electrodes were assessed for data preprocessing. Preprocessing was performed with BESA (www.besa.de). EEG data were re-referenced to the mean reference and filtered with a 0.10 Hz high-pass filter (6 dB/octave) and a 40 Hz low-pass filter (24 dB/octave). Segmentation was performed from 100 ms before image onset (baseline) to 1000 ms after stimulus presentation. Event-related potentials were analyzed. They were time-locked to the presentation of the faces. Eye movements were corrected using BESA’s automatic eye artifact correction [53]. Bad channels were identified by visual inspection and interpolated. As some participants (*n* = 6) did not have acceptable data (conditions with less than 60% accepted trials), they were excluded from further analyses and were not part of the final 60 participants. On average, 4.1% of all sensors were interpolated. For the included subjects, on average, 0.03% of all trials were marked as artifacts and therefore excluded from further analyses. Electrodes were selected on the basis of maximum activity during the inspection of topographic maps with EMEGS (http://www.emegs.org, [54]). In addition, the time windows used were selected based on visual inspection of the data. Thus, for the P100, the time window 70 - 110ms and a cluster of 10 occipital electrodes (PO5, PO7, PO9h, P7h, P7, PO6, PO8, PO10h, P8, P8h) were used. P200 amplitudes were obtained in the 160 - 240 ms time window using nine occipital electrodes (OI1, O1, PO3, POOz, Oz, OIz, OI2, O2, PO4). Since both angry and disgusted faces are considered relevant in the context of peer victimization, the mean amplitude for angry and disgusted faces was calculated (anger/disgust trials). Thus, the EN-trials consisted of anger/disgust trials, sadness trials, and happiness trials. Outliers were replaced using the winsorizing method [55] for each person’s mean amplitude for each ERP component and valence of the face stimuli. Mean amplitudes that deviated more than three standard deviations from the mean amplitude were replaced by the highest or lowest possible value within this range for each component and valence.

### Statistical analyses

All statistical analyses were performed using R version 4.2.2 [56]. Sample sizes for multiple regression analyses were estimated using G*Power [57]. With an assumed medium to large effect size (Cohen’s f^2^ = 0.25; [58]), with $\alpha$ = 0.05, power = 0.95, and the planned inclusion of four predictors (age, gender, child maltreatment, and peer victimization), the estimated required sample size was 55 participants. Hierarchical regression models were calculated to analyze the unique influence of peer victimization experiences on the various index scores and mean P100 and P200 amplitudes. A *p*-value < .05 was considered significant for all analyses. In addition, all correlation analyses, and hierarchical regression analyses were adjusted using the false discovery rate (FDR) correction [59]. In addition to age, the total score on the CTQ was included in the first step to control for its influence on the various outcomes. As gender had no effect on the results, only the models without gender are reported. Models were also calculated using the total score of the STAI and the SCL-27 as a predictor. The predictors were not significant (all *p* > .05) and are therefore not reported. In the second and final step, the FBS total score was included in the model. Three different trial types were analyzed (anger/disgust trials, sadness trials, and happiness trials). There was no violation of the multicollinearity assumption (all tolerances $\ge$ 0.75, all variance inflation factors $\le$ 1.33). In addition, to analyze the unique cortical responses to the different emotional stimuli when significant associations were present, standardized residuals were calculated for each emotion, taking into account the physiological response to neutral stimuli, as this approach is considered more reliable than difference scores [60–62]. Therefore, separate regression analyses were calculated with the mean amplitude in neutral trials as the variable predicting the mean amplitude in anger/disgust, happiness, and sadness trials. Pearson correlations were calculated between the standardized residuals and the FBS total score.

## Results

### Behavioral Data

Means and standard deviations for the different trial types and index scores are presented in Table 2. In addition, Pearson correlation coefficients between peer victimization experiences and the three index scores can be found for each trial type in Table 3, showing no significant correlation between behavioral data and peer victimization experiences (all *p*’s > .05). The hierarchical regression analyses for anger/disgust trials showed no significant influence of peer victimization experiences on the attentional bias score ($\beta$ = -0.12, *p* = .400; final model: *F* (3,56) = 1.26, adjusted *R*$^2$ = .01, *p* = .296), orienting score ($\beta$ = -0.20, *p* = .157; final model: *F* (3,56) = 3.16, adjusted *R*$^2$ = .10, *p* = .032), and disengaging score ($\beta$ = 0.09, *p* = .540; final model: *F* (3,56) = 0.81, adjusted *R*$^2$ = .00, *p* = .495). Similarly, there were no significant effects of peer victimization on attentional bias scores, orienting scores, and disengaging scores for sadness and happiness trials (for the regression analyses of the behavioral data, see Tables A1, A2, and A3 in the Supplementary Material).

**Table 2:** Means and standard deviations for the different trial types and index scores

Index score	Anger/Disgust	Happiness	Sadness
Attentional score	-1.94 (11.00)	-0.42 (17.11)	0.30 (21.31)
Orienting score	-1.75 (11.27)	-1.65 (13.13)	-0.63 (15.96)
Disengaging score	-0.19 (9.83)	1.24 (14.47)	0.93 (12.75)

**Table 3:** Pearson correlation coefficients of peer victimization and the different index scores for each trial type

Index Score	*r* peer victimization^a^	*p*	*p*_*FDR*_
Attentional bias score			
Anger/Disgust	-.01	.969	1.000
Sadness	-.04	.748	1.000
Happiness	.06	.649	1.000
Orienting score			
Anger/Disgust	-.06	.642	1.000
Sadness	-.08	.549	1.000
Happiness	-.03	.804	1.000
Disengaging score			
Anger/Disgust	.06	.624	1.000
Sadness	.03	.831	1.000
Happiness	.04	.754	1.000

*Note.* *p*_*FDR*_ values are FDR-adjusted ^a^Fragebogen zu belastenden Sozialerfahrungen (Adverse Social Experiences Questionnaire)

### ERP

**Fig. 2:**
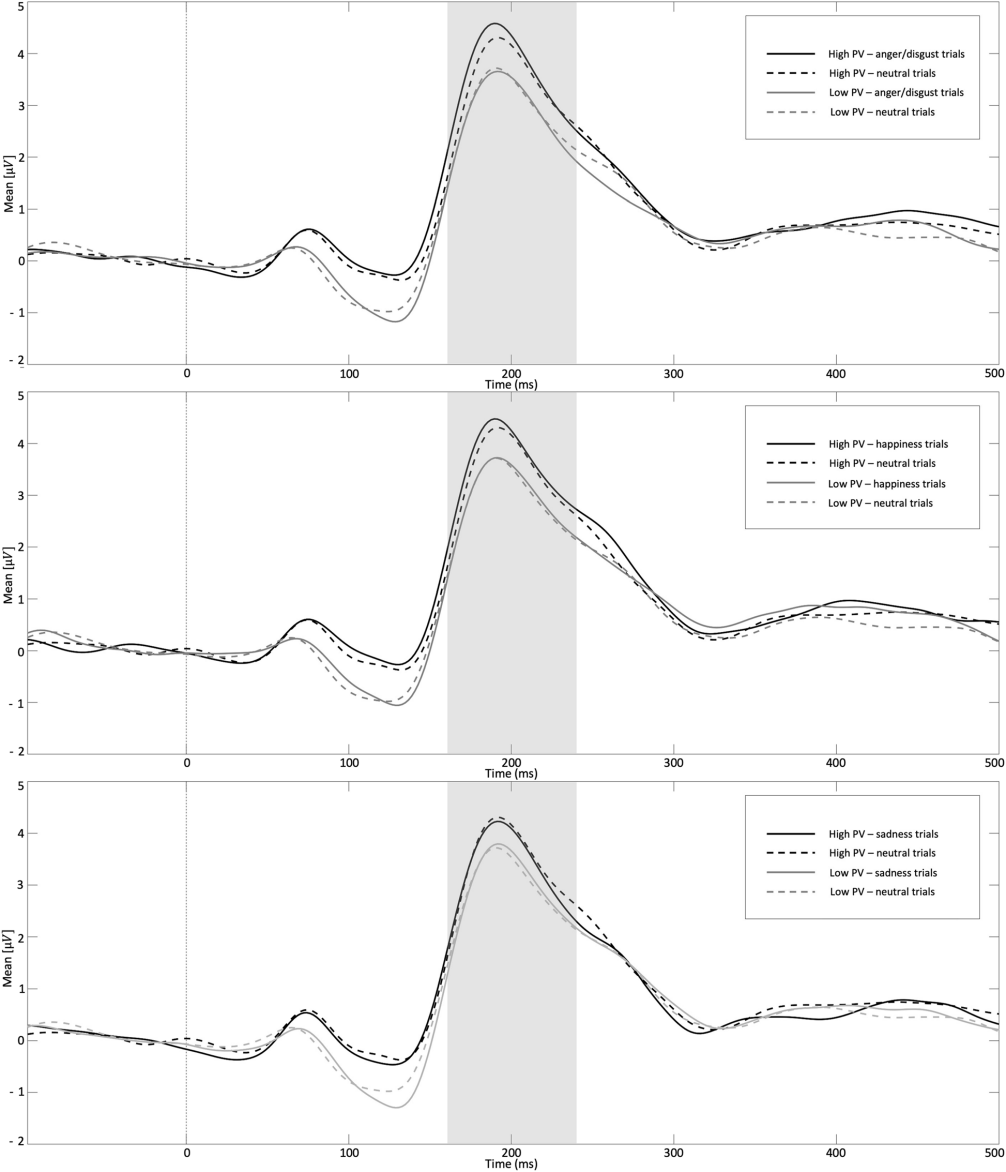
P200 mean amplitude (with electrode cluster OI1, O1, PO3, POOz, Oz, OIz, OI2, O2, PO4) for the different trial types. To illustrate the relationship with peer victimization, this predictor was dichotomized (median split)

The mean P100 and P200 amplitudes and standard deviations for each valence are shown in Table 4. In addition, correlation analyses of peer victimization experiences and mean P100 and P200 amplitudes for each trial type are shown in Table 5. The correlation analyses revealed a significant positive correlation between peer victimization and P200 amplitude in anger/disgust, happiness, sadness, and neutral trials (see Fig. 2).

**Table 4:** P100 and P200 mean amplitudes for the different valences (*M* (*SD*))

	Anger/Disgust	Happiness	Sadness	Neutral
P100	0.90 (1.26)	0.78 (1.23)	0.85 (1.40)	0.75 (1.20)
P200	3.16 (1.99)	3.08 (1.86)	3.20 (2.00)	3.08 (1.93)

*Note.* Means and standard deviations refer to the winsorized values

**Table 5:** Pearson correlation coefficients of peer victimization and the P100, and P200 amplitudes to all valences, (*N*=60)

	*r* peer victimization	*p*	*p*_*FDR*_
P100			
Anger/Disgust	-.06	.626	1.000
Happiness	-.02	.880	1.000
Sadness	.04	.761	1.000
Neutral	-.11	.395	1.000
P200			
Anger/Disgust	.35 **	.006	.024
Happiness	.28*	.029	.032
Sadness	.31*	.016	.039
Neutral	.28*	.031	.031

*Note.* *p<0.05, **p<*0.01*; *p*_*FDR*_ values are FDR-adjusted

### P100

Hierarchical regression analyses for the mean P100 amplitude are shown in Table 6. The regression models for anger/disgust trials (final model: *F* (3,56) = 1.54, adjusted *R*$^2$ = .03, *p* = .213), sadness trials (final model: *F* (3,56) = 2.68, adjusted *R*$^2$ = .08, *p* = .055), and happiness trials (final model: *F* (3,56) = 0.74, adjusted *R*$^2$ = .00, *p* = .535) showed no significant relationship between peer victimization experiences and P100 mean difference amplitude.

**Table 6:** Hierarchical multiple regression analyses for the mean P100 amplitude for the different trial types

	$\varvec{\beta }$	*R*^2^	adj. $\varvec{R}^{\varvec{2}}$	$\varvec{\bigtriangleup }$ $\varvec{R}^{\varvec{2}}$	*F*	*p*	*p*_*FDR*_
**Anger/disgust trials**							
Step 1		.07	.04	.07	2.09	.133	
Age	.28*					.042	.126
Child maltreatment	-.04					.798	.798
Step 2		.08	.03	.01	1.54	.213	
Peer victimization	-.10					.486	.729
**Happiness trials**							
Step 1		.04	.00	.04	1.06	.354	
Age	.20					.151	.453
Child maltreatment	-.01					.927	.927
Step 2		.04	.00	.00	0.74	.535	
Peer victimization	-.05					.723	1.000
**Sadness trials**							
Step 1		.12	.09	.12	4.03*	.023	
Age	.35*					.010	.030
Child maltreatment	.05					.755	.755
Step 2		.13	.08	.01	2.68	.055	
Peer victimization	-.05					.736	1.000

*Note.* *p<*0.05*; *p*_*FDR*_ values are FDR-adjusted; $\beta$ coefficients correspond to those of the final model

### P200

Hierarchical regression analyses of P200 amplitude for the different trial types are presented in Table 7. As shown, the results revealed a significant association between P200 amplitude and peer victimization in anger/disgust trials. However, the overall regression model was not significant (final model: *F* (3, 56) = 2.63, adjusted *R*$^2$ = .08, *p* = .059). There was no significant relationship between peer victimization experiences and P200 amplitude in happiness trials (final model: *F* (3, 56) = 1.69, adjusted *R*$^2$ = .03, *p* = .180). In addition, there was no significant effect in sadness trials (final model: *F* (3, 56) = 2.03, adjusted *R*$^2$ = .05, *p* = .120), although peer victimization was a significant predictor in this model. Following the significant associations between peer victimization and the P200 across trial types, correlation analyses of peer victimization and the standardized residuals revealed a significant correlation for anger/disgust trials. Higher levels of peer victimization were associated with a more pronounced P200 amplitude in anger/disgust trials when the electrocortical response to neutral faces was considered (see Table 8).

**Table 7:** Hierarchical multiple regression analyses for the mean P200 difference amplitude

	$\varvec{\beta }$	$\varvec{R}^{\varvec{2}}$	adj. $\varvec{R}^{\varvec{2}}$	$\varvec{\bigtriangleup }$ $\varvec{R}^{\varvec{2}}$	*F*	*p*	*p*_*FDR*_
**Anger/disgust trials**							
Step 1		.03	.00	.03	1.02	.368	
Age	-.04					.753	1.000
Child maltreatment	.04					.757	.757
Step 2		.12	.08	.09	2.63	.059	
Peer victimization	.34*					.021	.063
**Happiness trials**							
Step 1		.03	.00	.03	0.86	.430	
Age	-.04					.766	.766
Child maltreatment	.06					.671	1.000
Step 2		.08	.03	.05	1.69	.180	
Peer victimization	.26					.075	.225
**Sadness trials**							
Step 1		.03	.00	.03	0.94	.396	
Age	-.02					.881	.881
Child maltreatment	.05					.714	1.000
Step 2		.10	.05	.07	2.03	.120	
Peer victimization	.29*					.048	.144

*Note.* *p<*0.05*; *p*_*FDR*_ values are FDR-adjusted; $\beta$ coefficients correspond to those of the final model

**Table 8:** Pearson correlation coefficients of peer victimization and standardized residuals of P200 amplitudes in all trial types, (*N*=60)

	*r* peer victimization	*p*	*p*_*FDR*_
P200			
Anger/Disgust	.28*	.032	.096
Happiness	.05	.697	.697
Sadness	.13	.307	.461

*Note.* *p<*0.05*; *p*_*FDR*_ values are FDR-adjusted

## Discussion

Following on from previous findings of attentional biases related to peer victimization experiences and ambiguous and limited ERP findings in this context, the present study provides new insights into the behavioral and cortical responses to emotional facial expressions associated with peer victimization. The dot-probe task was used to analyze both behavioral (i.e., reaction times) and cortical (i.e., ERP) responses to emotional facial expressions of different valence. Correlation analyses and hierarchical regression analyses revealed a relationship between occipital P200 amplitudes and reported frequency of peer victimization experiences during childhood and adolescence. There was no relationship between reaction times and peer victimization experiences, or between the P100 amplitudes and peer victimization experiences.

The present results suggest a more pronounced occipital P200 amplitude in response to anger/disgust trials in participants with higher levels of relational peer victimization. Carretié et al. [33] showed a more pronounced P200 for negative stimuli preceded by a cue, suggesting sensitivity to negative visual information. Using source location analyses, the authors found the origin in the dorsal stream, which was found to be more activated in response to negative stimuli compared to neutral or positive stimuli [33]. The authors suggested that this activation is influenced by the action-relevant nature of negative stimuli. This specificity for negative cues seems to occur mainly in certain groups of individuals. Bar-Haim, Lamy, and Glickman [63] found a more pronounced occipital P200 amplitude in response to angry faces in anxious compared to non-anxious participants, which may indicate a greater amount of mobilized resources. Our results suggest a modulation of the P200 in the context of peer victimization experiences. This altered amplitude in response to potentially threatening and negative stimuli, suggesting a greater allocation of attentional resources to these stimuli, could be seen as a consequence of adaptive adjustment processes and the importance for motor activity [33]. These findings extend behavioral analyses of peer victimization, which already point to the presence of attentional biases for threatening stimuli [20]. In addition, the results suggest a more pronounced P200 amplitude in response to sadness trials in peer victimized participants. This is in line with findings of facilitated attention to sad facial expressions in individuals reporting higher levels of peer victimization [28]. In this study, facilitated attention of sad faces has been related to mood-congruency effects [15, 18, 64, 65] indicating the relevance of sadness in the context of peer victimization [28]. Notably, the relationship between peer victimization experiences and enhanced processing of negative social stimuli has been found in adults for whom the victimization experiences occurred years ago. In this context, the persistent alteration in cortical processes may be increasingly associated with costs, as the originally threatening stimuli no longer pose an immediate threat, and the distorted attention may lead to a biased perception of the environment [66]. Following on from findings of altered attentional processes in anxious individuals [63], these cortical changes may represent a risk factor for the development of psychopathology. Further longitudinal research and studies including participants with and without clinical diagnoses would be needed to further investigate the relationship between peer victimization experiences, altered cortical processes, and psychopathology.

In addition, the results partially indicate that a more pronounced P200 amplitude in happiness trials is associated with higher levels of peer victimization experiences. The results suggest that the increased attention is not only related to negative stimuli, but to facial stimuli in general. This is also consistent with the finding of a positive correlation of P200 amplitude even in neutral trials. This is in line with previous reports of generalized attention processing of negative and positive stimuli in peer victimized individuals [19]. However, analyses show that this effect disappeared when additional predictors were included. Since no effects were found in the regression model, this finding should be interpreted with caution. It should be noted that the *p*-values in the regression analyses and in the correlation analyses analyzing the unique variance did not withstand the FDR correction and the overall models were not significant. In the multiple regression models, this is probably due to overfitting. However, the findings regarding potential threat are consistent across different analyses and are also evident when the cortical response to neutral stimuli was considered. Nevertheless, replication studies with larger samples are needed to confirm the findings.

Unexpectedly, there was no association between P100 amplitudes and the level of peer victimization experiences. Thus, the results are in contrast to the findings of Iffland et al. [26] and Pollak and Tolley-Schell [31], whose results indicate a modulation of P100 amplitude associated with maltreatment experiences, including peer-victimization. Pollak and Tolley-Schell [31] showed a more pronounced P100 amplitude in response to angry faces in maltreated children. As the P100 is primarily associated with very early basal processing and initial face processing [9], this finding, combined with our finding of increased P200 amplitude, suggests that peer victimization experiences modulated the cortical response later in this sample. The lack of a relationship with the processing of threatening stimuli may be related to the characteristics of the present sample. The level of experienced peer victimization in the present sample is lower than in comparable studies of peer-victimized adults (e.g., [20, 28]). The findings suggest that hypervigilance to threatening stimuli at this very early stage of processing is more likely to be found in individuals with higher levels of experienced peer violence. Furthermore, the stimuli used in the present experiment were not embedded in a specific emotional context. In contrast to the experimental design of Iffland et al. [26], only emotional faces were presented without any further information. It cannot be excluded that the experimental design influenced the results in terms of cortical response. In spite of the null findings in the present study, the results of the above-mentioned studies suggest that the P100 is a potential marker for the effects of peer violence in relationships and that peer violence affects very early stages of cortical processing.

Regarding behavioral measures, no relationship was found between peer victimization and reaction times. Thus, the behavioral measures point in a different direction than the cortical measures and also contradict the findings of Iffland et al. [19] and Iffland and Neuner [20], who were also able to find attentional biases at the behavioral level in individuals with peer victimization experiences. The results may suggest that reaction time analysis is not sensitive enough to detect attentional biases in the present sample. Based on the EEG results, a bias in response to emotional facial expressions would have been expected. The stimuli used here are relatively basal compared to the dot-probe studies of Iffland et al. [19] and Iffland and Neuner [20], which presented emotional words or emotionally conditioned neutral faces. It is possible that these simple facial stimuli were not sufficient to reveal attentional biases at the behavioral level. Furthermore, the inconclusive results for ERP and reaction times are consistent with other studies showing divergent results for cortical and behavioral measures [63, 67]. These findings raise questions about the reliability of the dot-probe task in measuring attentional processes on a behavioral level [68]. In a dot-probe study by Kappenman et al. [67] the authors found no attentional bias when analyzing reaction time, but analysis of event-related potentials revealed an attentional bias to threat. The authors argued that ERPs are more likely to capture rapid shifts of attention and that behavioral response is not sensitive enough because there is more time between stimulus presentation and response to the target stimulus [67]. Future studies should use other paradigms for the behavioral analysis of attentional biases. It remains open whether the behavioral results are due to the fact that the enhanced processing does not affect behavior, that the stimulus material was not intense or action-relevant enough, or that the dot-probe experiment was not sensitive enough to detect differences at the behavioral level. The findings also highlight the need for replication studies, as the results suggest that the experimental design may play an important role.

Some limitations should be considered when interpreting the results. It should be noted that medication and the presence of mental illness was only partially considered in the analyses. A total of 15% of participants stated that they had experienced a mental illness. It cannot be excluded that this influenced the results. However, potential effects were reduced by adjusting the reaction time and EEG data and by excluding participants with current higher levels of depressive symptoms from the analyses.

Another limitation is that the level of peer victimization experience was lower than in comparable studies of peer victimization (e.g.,; [20, 28]). This limits the interpretation of the results and makes comparisons with other studies difficult. It cannot be excluded that the conflicting findings regarding behavioral and ERP outcomes are due to these factors. Future studies should include a more mixed sample, e.g., by including a group of patients.

For the analysis of the EEG data, trials in which subjects gave incorrect or delayed responses were included in the analyses. It cannot be excluded that this had an effect on the results. However, it can be assumed that this did not have a major impact, since the overall number of incorrect responses was low and an adjustment was made on the basis of outliers. Thus, it can be assumed that highly deviant cortical responses, e.g., due to not following the instructions, were excluded and did not influence the results.

Another limitation is that stimulus onset asynchrony (SOA) was not varied. Thus, the target was always presented after 500 ms, making it unclear whether initial attention had already shifted. However, since ERP analysis allows one to capture early attentional processes, conclusions can be drawn about the early stages of the processes. Nevertheless, future studies should vary the SOA to allow more precise statements at the behavioral level and to analyze the processes in a more differentiated way [69]. Furthermore, the retrospective recording of life experiences from childhood and adolescence remains susceptible to bias [70]. However, the FBS is a questionnaire with high reliability and validity [21]. In the present sample, the FBS also showed acceptable internal consistency and can therefore be considered suitable for retrospectively capturing experiences of peer victimization.

## Conclusion

Previous findings have suggested altered attentional processes in individuals with peer victimization experiences. The present study demonstrates altered physiological responses to emotional facial expressions in individuals with a history of peer victimization experiences. Specifically, individuals with higher levels of peer victimization experiences showed a more pronounced occipital P200 amplitude in response to facial expressions, especially in response to threatening faces. Thus, the results indicate an enhanced activation of attentional resources in response to negative stimuli. These findings suggest that peer victimization in childhood and adolescence may also alter cortical processes in the long term. The extent to which these changes may be related to the later development of psychopathology should be further investigated in future longitudinal studies.

### Supplementary Information

Supplementary Material 1.

## Data Availability

The datasets used and/or analyzed during the current study are available from the corresponding author on reasonable request.

## Abbreviations

ERP: Event-related potential
EEG: Electroencephalography
SCL-27: Symptom Check List-27
BDI II: Beck Depression Inventory II
STAI: State-Trait-Anxiety-questionnaire
FBS: Fragebogen zu belastenden Sozialerfahrungen (Adverse social experiences questionnaire)
CTQ: Childhood Trauma Questionnaire
