# Supplementary material for: Attentional processes in response to emotional facial expressions in adults with retrospectively reported peer victimization of varying severity: Results from an ERP dot-probe study
Source: BMC Psychol. 2024 Aug 29;12:459. doi: 10.1186/s40359-024-01958-5 (PMC11361057; doi:10.1186/s40359-024-01958-5)
Supplement: Supplementary file 1 — Supplementary Material 1. [file 40359_2024_1958_MOESM1_ESM.pdf]

Supplementary material

Table A1

Hierarchical multiple regression analyses for anger/disgust trials

Variable	β	R^2	adjusted R^2	$\triangle R^2$	F
Attentional bias score					
Step 1		.05	.02	.05	1.54
Age	-.07				
Child maltreatment	.29				
Step 2		.06	.01	.01	1.26
Peer victimization	-.12				
Orienting score					
Step 1		.11	.08	.11	3.64*
Age	-.23				
Child maltreatment	.40**				
Step 2		.14	.10	.03	3.16*
Peer victimization	-.20				
Disengaging score					
Step 1		.03	.00	.03	1.03
Age	.18				
Child maltreatment	-.14				
Step 2		.04	-.01	.01	0.81
Peer victimization	.09				

Note. $^*p<0.05$, $^{**}p<0.01$; β coefficients correspond to those of the final model.

Table A2

Hierarchical multiple regression analyses for happiness trials

Variable	β	R^2	adjusted R^2	$\triangle R^2$	F
Attentional bias score					
Step 1		.05	.01	.05	1.38
Age	.21				
Child maltreatment	.02				
Step 2		.05	.00	.00	0.91
Peer victimization	.01				
Orienting score					
Step 1		.04	.00	.04	1.06
Age	-.12				
Child maltreatment	.20				
Step 2		.04	-.01	.00	0.71
Peer victimization	-.03				
Disengaging score					
Step 1		.12	.08	.12	3.70*
Age	.35*				
Child maltreatment	-.16				
Step 2		.12	.07	.00	2.46
Peer victimization	.04				

Note. *$p<0.05$*; β coefficients correspond to those of the final model.

Table A3

Hierarchical multiple regression analyses for sadness trials

Variable	β	R^2	adjusted R^2	$\triangle R^2$	F
Attentional bias score					
Step 1		.09	.06	.09	2.77
Age	-.09				
Child maltreatment	.40**				
Step 2		.12	.07	.03	2.59
Peer victimization	-.20				
Orienting score					
Step 1		.13	.10	.13	4.19*
Age	-.25				
Child maltreatment	.43**				
Step 2		.17	.12	.04	3.78*
Peer victimization	-.23				
Disengaging score					
Step 1		.04	.01	.04	1.23
Age	.15				
Child maltreatment	.13				
Step 2		.04	-.01	.00	0.86
Peer victimization	-.06				

Note. *$p<0.05$, **$p<0.01$*; β coefficients correspond to those of the final model.
